# Chronic exposure to bisphenol A induces behavioural, neurochemical, histological, and ultrastructural alterations in the ganglia tissue of the date mussels *Lithophaga lithophaga*

**DOI:** 10.1007/s11356-023-29853-3

**Published:** 2023-09-28

**Authors:** Heba-Tallah Abd Elrahim Abd Elkader, Ahmed S. Al-Shami

**Affiliations:** 1 Zoology, Biological and Geological Sciences Department, Faculty of Education, Alexandria University, Alexandria, Egypt; 2 Zoology Department, Faculty of Science, Alexandria University, Alexandria, Egypt; 3 Biotechnology Department, Institute of Graduate Studies and Research, Alexandria University, Alexandria, Egypt

**Keywords:** BPA, *Lithophaga lithophaga*, Ganglia, Behaviour, Histopathology, TEM

## Abstract

Bisphenol A (BPA), a common plastic additive, has been demonstrated mechanistically to be a potential endocrine disruptor and to affect a variety of body functions in organisms. Although previous research has shown that BPA is toxic to aquatic organisms, the mechanism of neurotoxic effects in marine bivalves remains unknown. The current study aimed to elucidate the neurotoxic effects of BPA when administered at different concentrations (0.25, 1, 2, and 5 µg/L) for twenty-eight days in the ganglia of a bivalve model, the Mediterranean mussel (*Lithophaga lithophaga*), which is an ecologically and economically important human food source of bivalve species in the Mediterranean Sea. Our findings revealed an increase in behavioural disturbances and malondialdehyde levels in treated mussel ganglia compared to the control group. Furthermore, superoxide dismutase activity increased in the ganglia of *L. lithophaga* treated with 0.25 and 2 µg/L. However, at BPA concentrations of 1 and 5 µg/L, SOD activity was significantly reduced, as was total glutathione concentration. BPA causes neurotoxicity, as evidenced by concentration-dependent inhibition of acetylcholinesterase, dopamine, and serotonin. After chronic exposure to BPA, neurons showed distortion of the neuronal cell body and varying degrees of pyknosis. The ultrastructure changes in BPA-treated groups revealed the lightening of the nucleoplasm and a shrunken nuclear envelope. Overall, our findings suggest that BPA exposure altered antioxidation, neurochemical biomarkers, histopathological, and ultrastructural properties, resulting in behavioural changes. As a result, our findings provide a basis for further study into the toxicity of BPA in marine bivalves.

## Introduction

Recent estimates suggest that between 4.8 and 12.7 million Tons of plastic litter enter the world’s oceans each year (Jambeck et al. 2015; Beiras et al. 2018). Conventional plastic polymers are not biodegradable and are expected to last for hundreds of years in the environment. However, neither floating plastic debris (Law et al. 2014) nor sunk plastic debris (Galgani et al. 2015) show increasing temporal trends. Plastic objects become increasingly brittle and susceptible to mechanical abrasion as a result of thermo-oxidative breakage of the polymeric chains, photodegradation, and leaching of plasticizing additives, particularly in high-energy environments such as the exposed coastline (Beiras et al. 2018). Bisphenol A (BPA) is a chemical that is frequently used in the production of epoxy resins and polycarbonate plastics, both of which are the major sources of daily exposure (Flint et al. 2012; Abd Elkader et al. 2021). BPA, a well-known endocrine disruptor, is continuously discharged into aquatic environments, resulting in a ubiquitous presence of BPA in aquatic invertebrates at greater concentrations in freshwater systems than in marine and estuarine systems (Sheir et al. 2020; Wu and Seebacher 2020). BPA is a toxin that has been linked to changes in lipid peroxidation, endocrine function, and the production of reactive oxygen species in both vertebrate and invertebrate organisms (Benjamin et al. 2019; Abdou et al. 2022). The bulk of BPA aquatic toxicity data from multiple sources reveal EC10, EC50, and LC50 values for microorganisms, invertebrates, and fish that are greater than 1000 µg/L (Hendriks et al. 1994). BPA has negative endocrine-disruptive effects in aquatic organisms at concentrations as low as 1.75 µg/L in Brown trout (Lahnsteiner et al. 2005), 1–25 µg/L in mud snails (Jobling et al. 2003), and 50 µg/L in mussels (Ortiz-Zarragoitia and Cajaraville 2006). According to the available literature, BPA surface water concentrations range from 0 to 56 µg/L (Corrales et al. 2015) around the world, and in sediment from 5.32 to 15.52 µg/kg in the Adriatic Sea (Andelic et al. 2015). The researchers examined sand and seawater from over 200 locations in 20 nations, primarily in Southeast Asia and North America. All had a “significant” quantity of BPA in them, ranging from 0.01 to 50 µg/L. BPA levels were found to be in a narrow range of 8.85–14.76 µg/L in the Black Sea, Bosphorus, and Sea of Marmara in Turkish city, Istanbul. Similarly, the Mediterranean Sea survey showed that BPA concentrations in surface seawater lie in a narrow range from 13.80 to 15.34 µg/L (Ozhan and Kocaman 2019). From July 2018 to April 2019, total and individual phenol concentrations in Alexandria coastal waters from 10 different locations between Abu Qir Bay and Eastern Harbour ranged from 30.33 to 47.07 ng/L. Thus, the levels observed in our seas appear to be higher than those that can potentially cause harmful endocrine-disruptive effects in marine species (Abd Elkader and Al-Shami 2023). The elevated BPA contents could suggest major plastic contamination in the Mediterranean.

The bivalve *Lithophaga lithophaga* is a species of economic importance as a popular food item among locals, particularly lactating mothers because it contains a significant amount of vitamin B12 and has a high percentage of protein, n-3 polyunsaturated fatty acids, and essential minerals when compared to other shellfish (Aakre et al. 2019; Abd-Ellah et al. 2020). Although the biomonitoring abilities of *L. lithophaga* due to its filter-feeding behaviour, sessile status, and capacity to concentrate pollutants to several orders of magnitude above ambient levels in aquatic environments, bibliographic data only provides limited information about the histological and ultrastructural effects of BPA on the neural cells (including neurosecretory) (Oliveira et al. 2014). Furthermore, growing evidence suggests that aquatic species’ brain systems may be the primary targets for waterborne BPA (Gagné et al. 2011; Saili et al. 2012; Kim et al. 2020). Although invertebrates are the most numerous marine organisms and play critical roles in marine ecosystems, the possible neurotoxic effects of BPA on marine invertebrates are yet unknown (Tang et al. 2020). Although *L. Lithophaga* has been used as a model species to investigate the ecotoxicological risks associated with various contaminants in invertebrates, the neurotoxicity of BPA in *L. Lithophaga* has not been elucidated. As a result, gaining a better understanding of its neurological system is advantageous for both comparative and practical purposes (Cuvillier-Hot and Lenoir 2020).

**Fig. 1:**
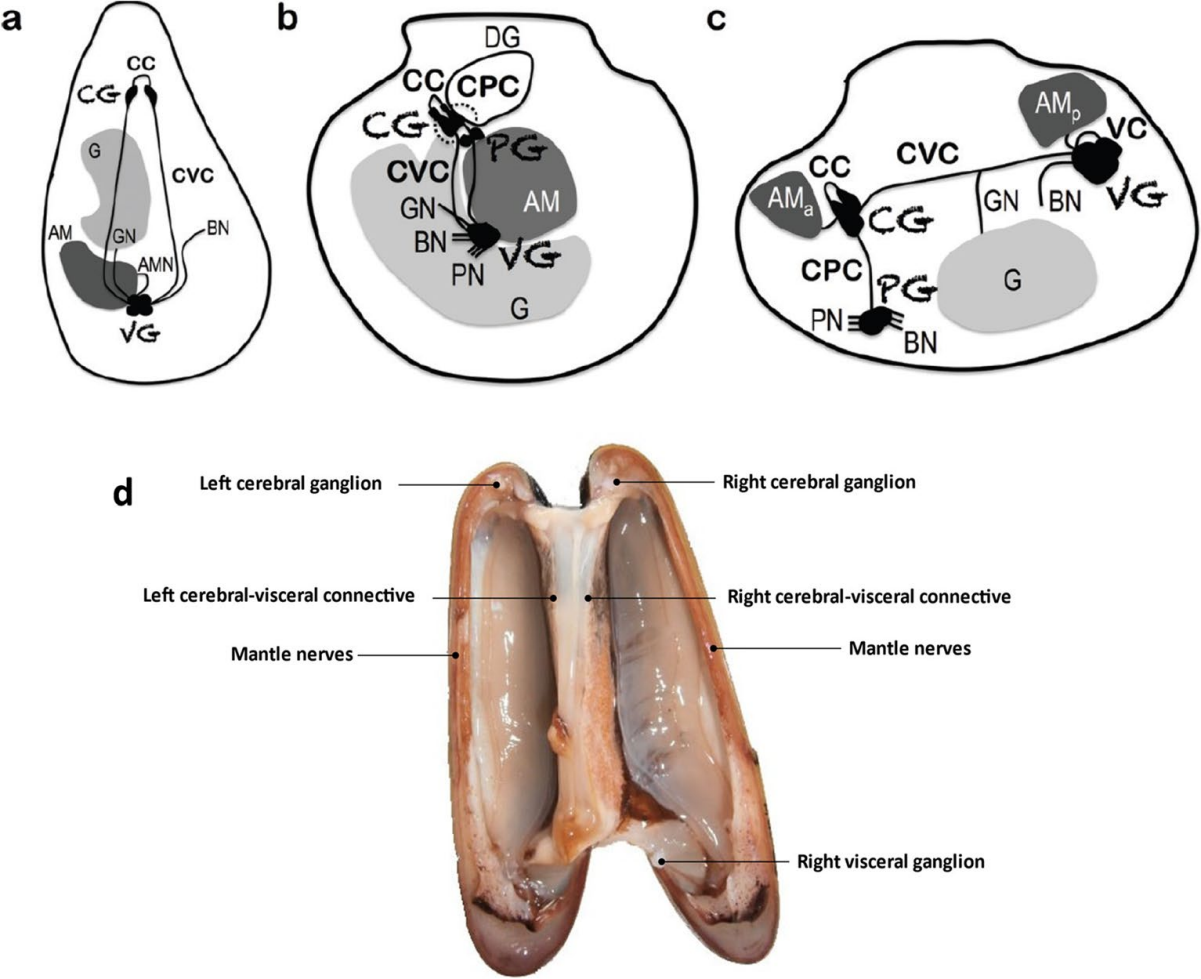
**a**–**c** Schematics of intercommunicating ganglia in Pacific oyster, *Crassostrea gigas* (**a**), Yesso scallop, *Patinopecten yessoensis* (**b**), soft-shell clam, *Mya arenaria* (**c**), respectively. It is decentralised, with bilaterally symmetrical cerebral ganglia (CG), pedal ganglia (PG), and visceral ganglia (VG). The locations of ganglia vary greatly between species; however, they are linked by nerve connectives. VG innervates the gonad in most bivalves. Anterior and posterior adductor muscle (AMa and p); adductor muscle nerves (AMN); bronchial nerves (BN); cerebral commissure (CC); cerebral-pedal connective (CPC); cerebral-visceral connective (CVC); digestive gland (DG); gonad (G); gonad nerves (GN); pallial nerve (PN) (Alavi et al. 2017). **d** The nervous system anatomy of the date mussel (*L. lithophaga*). It is decentralized and composed of bilaterally symmetrical cerebral ganglia (CG) and visceral ganglia (VG) linked by cerebral-visceral connectives

The nervous system (NS) of marine organisms is made up of specialised and organised cells (with specific shapes and functions) that work together to control the organism’s behaviour as well as sense and respond to environmental stimuli (Northcutt et al. 2017). Bivalves have a bilateral central nervous system (CNS), and fusion has typically reduced it to three distinct, large ganglia. The anterior cerebropleural ganglia produce two sets of nerve cords, one ventral to the pedal ganglia and the other posterodorsally to the visceral ganglia. Located over the oesophagus, a dorsal commissure connects the two cerebropleural ganglia. The palps, anterior adductor muscle, and mantle receive nerves from the cerebropleural ganglia (Fig. 1). The stomach, heart, gills, mantle, syphon, and posterior adductor muscles receive nerves from the visceral ganglia (Wanninger 2015). Invertebrate nervous systems are made up of two basic types of cells: neurons (mostly motor, sensory, and interneurons) and glial cells (Harrison and Kohn 1994; Tantiwisawaruji et al. 2017). Invertebrate neural cells have simple numerical proportions, a diversified composition and organisation, and large neurons (Meinertzhagen 2017). They are in charge of the electrical signalling transmission (or synapses) between them and among different body parts. Synapses are present in both vertebrates and invertebrates; with common mechanisms such as changes in ion concentrations and transmitter release (Watson 1992; Ortega and Olivares-Bañuelos 2020). On the other hand, glial cells participate in the early development of the NS, as well as in the support and protection of neurons, the maintenance of axon function for normal neurotransmission, the proportionate supply of metabolic fuel to neurons, and the homeostatic regulation of the NS (Brand and Livesey 2011). Furthermore, glial cells function as a blood–brain barrier and in the guiding processes of immature neurons to ensure their proper migration to specified locations. The number, kind, and placement of glial cells in invertebrates’ NS are variable. Glial cells in certain molluscs are small (4–5 µm in diameter) and their filaments are 0.2–0.5 µm thick, yet in others, such as the giant squid, glia cells are 100–600 µm long, 2 µm thick, and 20 µm wide (Coles 2009; Ortega and Olivares-Bañuelos 2020).

Invertebrates are no longer thought to have a canonical endocrine system, but rather a neuroendocrine system that regulates and develops through the secretion of neuropeptides and neurotransmitters (Malagoli and Ottaviani 2017; Joyce and Vogeler 2018). A neurosecretory/neuroendocrine system appeared very early in metazoan history so that all invertebrates possess neuroendocrine cells releasing neuropeptides in the haemolymph or coelomic fluid; moreover, in many invertebrates such as polychaetes and molluscs, many neurons, although not organised in a gland-like structure, were proved to be neurosecretory cells (Tessmar-Raible 2007; Miglioli 2021). Many studies on endocrine disruption in aquatic invertebrates have recently focused on gastropod molluscs (Oehlmann et al. 2007; Bannister et al. 2013; Leonard et al. 2014). BPA is toxic because it inhibits the acetylcholinesterase enzyme (AchE), which catalyses the hydrolysis of the neurotransmitter acetylcholine (Ach) after it is released at the nerve synapse (Li et al. 2022; Reynoso et al. 2022). Several studies have found that reductions in AchE activity are associated with behavioural effects that may impair the survival of exposed organisms (Umar and Aisami 2020; Deidda et al. 2021; Amer et al. 2022; Munshi et al. 2022; Shiry et al. 2023). Dopamine (DA) and serotonin (5-HT), which operate as neurohormones in bivalves as well as other invertebrates, are known to act in a variety of tissues and organs as either neurotransmitters or neuromodulators of various processes (Bullock and Horridge 1965; Tantiwisawaruji et al. 2017). 5-HT has been identified as a key neurotransmitter/neurohormone mediating several processes, including brain development, the ciliary beating of the gills, and neuromuscular control of the heart and the syphon (Siniscalchi et al. 2004). Recent research has revealed that either direct exposure to microplastics (Crump et al. 2020) or microplastic leachates (Seuront 2018) can disrupt the cognitive system, sensory perception, and consequently behaviour of marine invertebrates. For example, the intertidal mussel *Mytilus edulis* responds to microplastic leachates by increasing its aggregation rate and frequency (Seuront et al. 2021). Changes in the DA level, together with disruptions in their production and metabolism in the mollusc CNS, can start pathogenic processes and interfere with their behaviour and locomotor responses (Sakharov and Salanki 1982; Kotsyuba 2011).

Behaviour is an organism-level phenomenon that represents an acute cumulative effect and reflects the integration of exposure circumstances. There is relatively little research on the effects of BPA on behaviour in the *Lithophaga* mussel, despite behaviour’s emergence as a significant indicator in environmental toxicology. So, this work aimed to use a multi-biomarker approach in terms of neurotoxicity, oxidative stress response, and changes in behaviour, histopathology and ultrastructure to study the toxicological effects of BPA in the cerebral and visceral ganglia of the date mussel, *Lithophaga lithophaga*, in a 28-day experiment.

## Materials and methods

### Chemicals

All the reagents were of analytical grade. BPA [2,2-bis (4-hydroxy phenyl) propane (99%; Sigma-Aldrich, USA)] was dissolved in seawater to the desired concentration. After that, the solutions were exposed to ultrasonic treatment for 4 h and mechanical vibration overnight to ensure full dissolution (Mihaich et al. 2009). The solutions were continuously stirred until no undissolved material remained and were freshly prepared twice a week.

### Collection and maintenance of test organisms

Date mussels (*Lithophaga lithophaga*) with shell lengths of 5.50 ± 0.85 cm were collected in January 2022 at Elanfoshy Bay in Alexandria, Egypt (lat. 31.205753 N and long. 29.924526 E). Two hundred mussels were carefully collected and transferred to a cool box filled with seawater. Within 3 h, the animals were transported to the laboratory at El-Max Station for Applied Research, part of the National Institute of Oceanography and Fisheries. The mussels were acclimatised in the laboratory for 1 week before the toxicity test. During acclimatization, the mussels were kept at 20 °C in a glass aquarium (length/weight/height 55 × 28 × 33 cm) with 50 L of aerated, filtered seawater with a 12 h: 12 h photoperiod. The bottoms of the aquaria were coated with 1 cm of coarse sand. Furthermore, every 2 days, a filtration system for waste removal, constant zooplankton supply of *Nannochloropsis oculata* and replacement, and water quality testing was performed.

### Experimental design

Following acclimatization, thirty mussels were randomly placed in temperature-controlled (18 ± 2 °C) aerated glass tanks filled with a 3 L test solution, with three replicates of ten mussels per concentration labelled as control, BPA 0.25, BPA 1, BPA 2, and BPA 5*.* According to Park et al. (2018) and Abd Elkader and Al-Shami (2023), the control set-up contained only depurated water and sand, and the experimental set-ups contained depurated water, sand, and 0.25, 1, 2, and 5 µg/L of BPA, respectively. The mussels’ biometric measurements were as follows: length (5.50 ± 0.85 cm), width (2.29 ± 0.25 cm), and weight (12 ± 3 g). Every 2 days, the preferred concentrations were consciously dosed into the water tanks, and the water was changed using a semi-static method with a 100% water change. The experimental period lasted 4 weeks, during which proper maintenance measures were implemented. Water quality parameters (pH, temperature, dissolved oxygen, conductivity, and salinity) were measured before and after the test solutions were renewed (Rice et al. 2012). Tanks were checked daily for dead mussels (obviously gaping shells), but no mortality was observed for any exposures.

### Behaviour analysis

On the 20th day of the experiment, 12 mussels from each concentration were removed, and behavioural experiments were conducted. BPA concentrations of 0.25, 1, 2, and 5 µg/L were used to study behavioural changes in *L. lithophaga*. Corresponding control sets were also kept in water with no added BPA. There were 5 observations on 12 different individuals, each of which was recorded continuously for 30 min per observation for 5 days. There was a 5-min interval between consecutive observations. The observations were made both visually and through video recordings. During the experiments, neither food nor aeration was provided (Yasmeen et al. 2012). For monitoring, the following behavioural parameters were chosen: (i) locomotor behaviour as the number of times movement (Mov) in the form of gliding and turning occurred during the 30-min monitoring period; (ii) duration of extensive extensions of foot and syphons together (FSE) in minutes/30 min; and (iii) duration of complete valve closure (VC) in minutes/30 min.

### Ganglia tissue preparation

Mussels were collected at random and cerebral ganglia (CG) and visceral ganglia (VG) were dissected from each exposed group as well as the control at the end of the 28-day exposure for biochemical analysis, histological, and transmission electron microscope (TEM) examination.

### Ganglia tissue biochemical tests

Ten mussels (3 repetitions per concentration) from each group had their ganglia tissues minced and homogenised in 10 volumes of 50 mM ice-cold potassium phosphate buffer (pH 7.5), 0.1 mM EDTA, and 0.5% Triton X-100. The homogenate was centrifuged at 12,000 g for 15 min at 4 °C, and the supernatants were collected and stored at − 80 °C for biochemical analysis.

The protein content was calculated using the Lowry method and bovine serum albumin as a standard (Lowry et al. 1951). The results were given in milligrams per gram of tissue.

Lipid peroxidation was calculated using a colourimetric method based on thiobarbituric acid (TBA)-reactive substances and expressed in terms of malondialdehyde (MDA) content (Draper and Hadley 1990). A spectrophotometer was used to measure absorbance at 532 nm. MDA levels were calculated using a standard of 1,1,3,3-tetraethoxypropane and expressed as nanomoles per gram of tissue.

The superoxide dismutase (SOD) activity was measured using a standard commercial kit (EnzyChromTM Superoxide Dismutase Assay Kit; cat. no. ESOD-100, BioAssay Systems) according to the method of Magana-Cerino et al. 2020 Nawi et al. 2020 and Sahin et al. (2020). The results were given in units per milligram of protein.

Total glutathione was measured according to the DTNB-GSSG (5,50-dithio-bis-nitrobenzoic acid-glutathione disulphide) reductase recycling assay (Anderson 1985). Glutathione reductase, DTNB, and NADPH were present in the reaction mixture. The formation of 5-thio-nitrobenzoic acid at 412 nm followed the rate of reaction. Total glutathione concentration was calculated using a standard curve and expressed in µm glutathione per gram of tissue.

The AchE activity in the ganglia was measured using thiocholine quantification, as described by Ellman et al. (1961), using commercial kits obtained from Sigma-Aldrich (cat. no. MAK119) and following the manufacturer’s instructions. The enzyme activity was monitored for 5 min after the substrate concentration was added. The rate of the enzymatic reaction was measured spectrophotometrically at 412 nm in comparison to a blank without substrate. AchE activity was measured in nanomoles per minute per milligram of protein.

DA (cat. no. KA3838) and 5-HT (cat. no. LS-F4120) levels in the ganglia were determined using commercial ELISA kits obtained from Abnova and LifeSpan Biosciences, Inc., respectively. The optical density was measured in a microplate photometer in 15 min using the manufacturer’s protocol at 450 nm.

### Histological examination

After 28 days, five *L. lithophaga* were chosen at random from each of the five setups. Prying open the shells and inserting a toothpick between the valves prepared the specimen for histological analysis. The mussels were euthanized, and their CG and VG were carefully excised before being fixed for 24 h in 10% buffered formaldehyde. The tissues were immediately transferred to vials containing 70% ethanol for histological processing. The tissues were processed, paraffin-embedded, sectioned at 5 µm, stained with hematoxylin–eosin (H&E), and examined under an Olympus light microscope (Olympus LC20, Germany). The percentage of aberrations was calculated using an index modified by Costa et al. (2013). The semi-quantitative analysis was obtained by counting at least five replicates from a randomly selected area on each animal’s section.

### TEM examination

Cerebral and visceral ganglia (1 mm^3^, *n* = 3 for each) were fixed in buffered glutaraldehyde, followed by the 1% osmium tetroxide fixation. After that, the samples were dehydrated in a series of ethanols (50–100%) before being embedded in epoxy resin capsules. The capsules were placed in a 60 °C oven for 48 h. After hardening, the blocks were trimmed and sectioned for semi-thin-section examinations with a LEICA Ultra Cut UCT microtome. Ultrathin sections at 60–90 nm thick were cut and placed on copper grids. After drying overnight, the sections were stained with uranyl acetate and lead citrate. Finally, grids were examined and photographed with the appropriate magnifications at the Faculty of Science, Alexandria University, Egypt, using a JEOL JEM-1400 Plus Transmission Electron Microscope (Japan). Electron micrographs were obtained, the length and number of mitochondria, changes in mitochondrial and nuclear diameter (µm), and changes in surface area (µm) of mitochondria and nucleus, respectively were analysed morphometrically in five photos for each group, using the image analysis software (Image J, version 1.52e).

### Statistical analysis

All values were expressed as mean ± SE. All data were first checked for normality (Shapiro-Wilks test) and variance homogeneity (Levene’s test), then a one-way analysis of variance (ANOVA) and Tukey’s post hoc test was used to see if there was a significant difference between mussels in different groups for parametric data. However, the statistical significance levels in non-parametric data were determined by Kruskal–Wallis, followed by the Mann–Whitney *U* test. A *p*-value of 0.05 was considered statistically significant. SPSS version 16 was used for all analyses.

## Results

### The water quality of the test medium

The physicochemical properties of the test medium (pH, temperature, dissolved oxygen, conductivity, and salinity) were all measured in seawater as follows: temperature (17–21 °C), salinity (38.8–42.3%), pH (7.2–7.6), conductivity (58.4–62.9 ms/cm), and dissolved oxygen (8–9.6 mg/L).

### Behavioural indicators

Table 1 shows the values of the different behavioural markers in the *L. lithophaga* in the control and 0.25, 1, 2, and 5 µg/L BPA-exposed groups. Our findings revealed statistically significant differences in *L. lithophaga* behaviour (Mov, FSE, and VC) between the control and the 0.25, 1, 2, and 5 µg/L BPA-exposed groups. Mov and FSE were higher in the 0.25, 1, and 2 µg/L BPA-exposed groups compared to the control group. However, Mov and FSE completely stopped in mussels exposed to 5 µg/L BPA. The cessation of both Mov and FSE was initiated relatively late (Table 2). Mov was stopped in the first hour of day 1 in 5 µg/L BPA exposure, while FSE was stopped in the second hour of day 1 in 5 µg/L BPA exposure. The duration of VC in the 1 and 5 µg/L BPA-exposed groups is significantly longer than the control, with the 5 µg/L BPA-exposed groups having the longest durations. However, the duration of VC is insignificantly lower in the 0.25 and 2 µg/L BPA-exposed groups than in the control.

**Table 1:** The values of the different behavioural changes in control and the 0.25, 1, 2, and 5 mg/L BPA-exposed groups in the *L. lithophaga* clam

Groups	Behavioral markers		
	Mov	FSE (min)	VC (min)
Control	1.67 ± 0.14^a^	26.66 ± 1.48^a^	9.61 ± 1.12^ab^
BPA 0.25	3.25 ± 0.29^bcd^	29.33 ± 0.67^a^	8.75 ± 0.37^abd^
BPA 1	3.09 ± 0.26^bcd^	26.73 ± 1.64^a^	12.60 ± 1.50^ac^
BPA 2	3.42 ± 0.28^bcd^	28.45 ± 0.95^a^	8.60 ± 0.19^ad^
BPA 5	0.00 ± 0.00^e^	0.00 ± 0.00^b^	29 ± 1.00^e^

Values are means ± SE; *n* = 12 for each group. Statistically significant test for comparison was done by Kruskal–Wallis, followed by Mann–Whitney *U* test for non-parametric independent samples; different superscripts denote significant (*p* ≤ 0.05) differences between the means of a given variable in a column

*Mov* movement/30 min, *FSE* duration of extension of foot and siphon in min/30 min, *VC* duration of complete valve closure in min/30 min

**Table 2:** First cessation of movement (Mov) and foot-siphon extension (FSE) in *L. lithophaga*

Groups	First observed effect	
	Cessation of Mov	Cessation of FSE
Control	Not observed	Not observed
BPA 0.25	10th hour of day 5	8th hour of day 2
BPA 1	4th hour of day 4	9th hour of day 2
BPA 2	7th hour of day 3	1st hour of day 2
BPA 5	1st hour of day 1	2nd hour of day 1

### Neurotoxicity biochemical markers

**Fig. 2:**
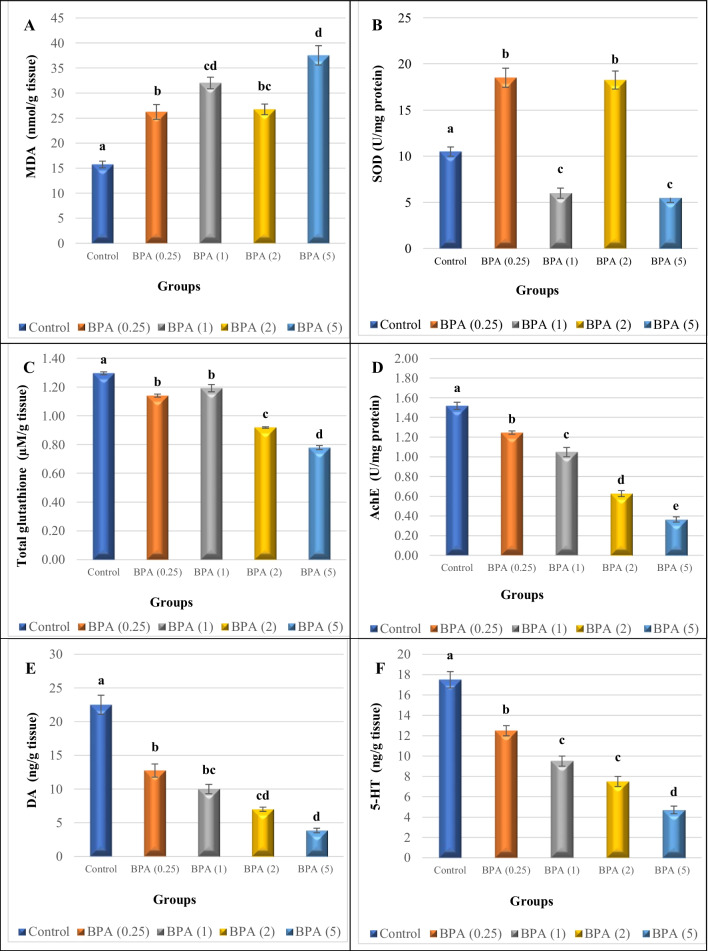
**A** Malondialdehyde (MDA) level (nmol MDA/g tissue); **B** superoxide dismutase (SOD) activity (U/mg protein); **C** total glutathione concentration (µm/g tissue); **D** acetylcholinesterase (AchE) activity (U/mg protein); **E** dopamine (DA); and **F** serotonin (5-HT) levels (ng/g tissue) in *L. lithophaga* in control and 0.25, 1, 2, and 5 µg/L BPA-exposed groups after 28 days. For each group, values are expressed as means ± S.E. (*n* = 5). The data were analyzed using one way ANOVA followed by Tukey’s test and statistical differences between the control and BPA-exposed treatments. Significant differences (*p* ≤ 0.05) between control and the four treatment concentrations are presented with letters (a–e)

Figure 2 summarises the biomarker results obtained from the chronic assay. The chronic bioassay revealed significant differences in lipid peroxidation, SOD and AchE activities, and total glutathione, DA, and 5-HT levels between treatments. When compared to the control, our findings revealed an increase in MDA levels (+ 67%, + 103%, 70%, and + 138%) in the ganglia of the BPA-treated groups, as shown in Fig. 2A.

In contrast, BPA exposure significantly (*p* ≤ 0.05) increased SOD activity (Fig. 2B) in the ganglia of *L. lithophaga* treated with 0.25 and 2 µg/L by 76% and 74%, respectively, when compared to controls. However, SOD activity was significantly reduced at BPA concentrations of 1 and 5 µg/L.

In comparison to the control group, BPA exposure in groups BPA 0.25, BPA 1, BPA 2, and BPA 5 resulted in significant (*p* ≤ 0.05) decrease (− 12%, − 8%, − 29%, and − 40%, respectively) in total glutathione concentration in *L. lithophaga* ganglia (Fig. 2C).

In comparison to controls, AchE activity in the *L. lithophaga* ganglia was reduced by 18%, 31%, 59%, and 76%, respectively, in BPA-treated groups (0.25, 1, 2, and 5 µg/L), during the treatment period (Fig. 2D).

Similarly, the levels of DA and 5-HT showed a similar trend, with a significant (*p* ≤ 0.05) reduction found between BPA treatments in comparison to the control (Fig. 2E, F).

### Macroscopic examination

The macroscopic examination of the nervous system network of *L. lithophaga* reveals bilateral symmetry in a sagittal plane regarding the animal’s main axis. It is made up of two pairs of white ganglia: CG and VG. The CG is positioned in the animal’s anterior area, above the oesophagus (near the labial palps), whereas the VG is located in the animal’s posterior area (in the centre of the adductor muscle). Connective nerves join each ganglion to the next, and inter-ganglionic nerve commissure connects them bilaterally (Fig. 1d). Cerebro-visceral connectives (CVC) are independent forms that connect with the CG and VG, respectively.

### Histological examination

**Fig. 3:**
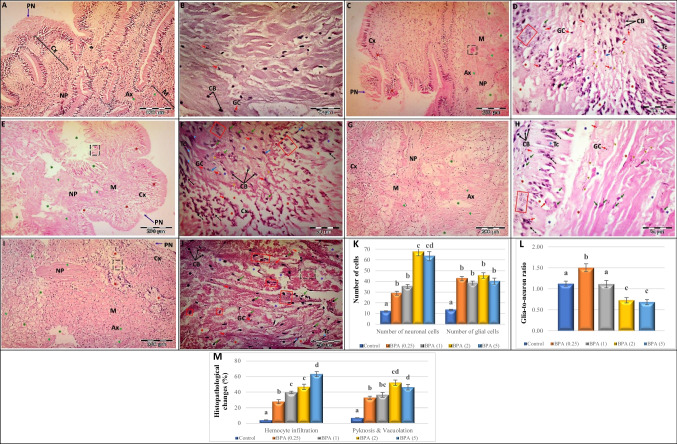
**A**–**J** Light micrographs showing transverse sections (T.S.) through the cerebral ganglia of *L. lithophaga* exposed to BPA stained with hematoxylin and eosin (H&E) illustrating the **A** and **B** control group, **C** and **D** 0.25 µg/L BPA-exposed group, **E** and **F** 1 µg/L BPA-exposed group, **G** and **H** 2 µg/L BPA-exposed group, **I** and **J** 5 µg/L BPA-exposed group. Abbreviations and symbols: cortex (Cx), medulla (M), neuronal cell body (CB) → , neuropile (NP), glial cell (GC)  , perineurium (PN)  , axon (Ax)  , hemocytes  , tear-drop cell (TC)  , apoptotic cell with perinuclear halo  , cells with acidophilic necrosis and pyknotic nuclei  , pericellular edema and vacuoles (  ), axonal degeneration (  ), hypercellularity of outermost cortical layer (black dashed arrow), degenerated neuron (red square), degenerated extracellular tissue (  ), hemocyte infiltration  . **K**–**M** Histopathological scores of cerebral ganglia of *L. lithophaga* exposed to BPA. **K** Number of neurons and glial cells in the cerebral ganglia of *L. lithophaga*. **L** Glia-to-neuron (number) ratio in the cerebral ganglia of *L. lithophaga*. **M** Histopathological changes (hemocyte infiltration, pyknosis, and vacuolation) in the cerebral ganglia of *L. lithophaga*. Values are expressed as means ± S.E. of 5 fields with 7 observations/group. Statistical analysis was done by ANOVA followed Duncan post hoc multiple comparison test. Significant differences (*p* ≤ 0.05) between control and the four treatment concentrations are presented with letters (a–e)

**Fig. 4:**
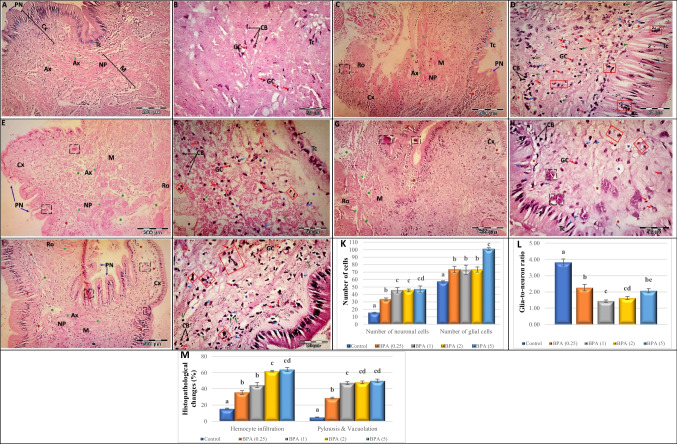
**A**–**J** Light micrographs showing transverse sections (T.S.) through the visceral ganglia of *L. lithophaga* exposed to BPA stained with hematoxylin and eosin (H&E) illustrating the **A** and **B** control group, **C** and **D** 0.25 µg/L BPA-exposed group, **E** and **F** 1 µg/L BPA-exposed group, **G** and **H** 2 µg/L BPA-exposed group, **I** and **J** 5 µg/L BPA-exposed group. Abbreviations and symbols: cortex (Cx), medulla (M), neuronal cell body (CB) → , neuropile (NP), glial cell (GC)  , perineurium (PN)  , axon (Ax)  , hemocytes  , tear-drop cell (TC)  , apoptotic cell with perinuclear halo  , cells with acidophilic necrosis and pyknotic nuclei  , pericellular edema and vacuoles (  ), axonal degeneration (  ), hypercellularity of outermost cortical layer (black dashed arrow), degenerated neuron (red square), degenerated extracellular tissue (  ), hemocyte infiltration  . **K**–**M** Histopathological scores of visceral ganglia of *L. lithophaga* exposed to BPA. **K** Number of neurons and glial cells in the visceral ganglia of *L. lithophaga*. **L** Glia-to-neuron (number) ratio in the visceral ganglia of *L. lithophaga*. **M** Histopathological changes (hemocyte infiltration, pyknosis, and vacuolation) in the visceral ganglia of *L. lithophaga*. Values are expressed as means ± S.E. of 5 fields with 7 observations/group. Statistical analysis was done by ANOVA followed Duncan post hoc multiple comparison test. Significant differences (*p* ≤ 0.05) between control and the four treatment concentrations are presented with letters (a–e)

Microscopic examination of sections from control mussels (Figs. 3A and 4A) showed the normal histological structure of the cerebral and visceral ganglia. Each of the paired CG and VG is covered by a thin layer of loose connective tissue in the periphery, known as the “perineurium”. Each ganglion is made up of a central nucleus known as a “neuropile”, which contains numerous tight nerve fibres and is surrounded by cells of all sizes and a small number of glial cells, which together constitute a structure known as the cortex. Three distinct cell types were observed in the cortical layer of both types of ganglia: (i) teardrop cells, (ii) elongated, and (iii) triangular cells. Neuronal cells appeared in the medulla as well, albeit infrequently. Furthermore, the musculature of the posterior adductor muscle, innervated by VG nerve fibres showed normal surface adhesion between the muscle bundles.

The histopathological changes in BPA-treated groups revealed different aspects in a concentration-dependent manner. Large neurons (teardrop cells) exhibited eccentric nuclei and severe chromatolysis in mussel CG and VG exposed to 0.25 µg/L BPA (Figs. 3 and 4C, D). The cytoplasm of numerous small neurons, on the other hand, was discovered to be dissolute and to have a visible perinuclear halo, while some other small neurons had acidophilic necrosis and pyknotic nuclei. Other small cell bodies shrank significantly and showed further heterochromatic staining of their nuclei. Furthermore, active microglial cells were discovered at various spots among neurons, and the extracellular space was observed enlarged and disorganised in some positions. Degenerated and apoptotic neurons of various sizes were also seen, as well as hemocyte infiltration and degeneration in nerve fibres generating axons that unite to create the neuropile and root.

The large and small neurons showed different stages of degeneration and severe pyknosis after chronic exposure to 1 µg/L BPA (Figs. 3 and 4E, F), including shrinkage of the cell body, migration of deformative nuclei, and hypercellularity of large neurons, in addition to degenerated and disorganised nerve fibres in the neuropile and root, as well as disorganisation of the perineurium. Furthermore, some neurons underwent total degeneration and apoptosis, as well as excessively enlarged and disorganised extracellular space, the presence of pericellular oedema and vacuoles, and the distribution of glial cells and hemocytes.

**Fig. 5:**
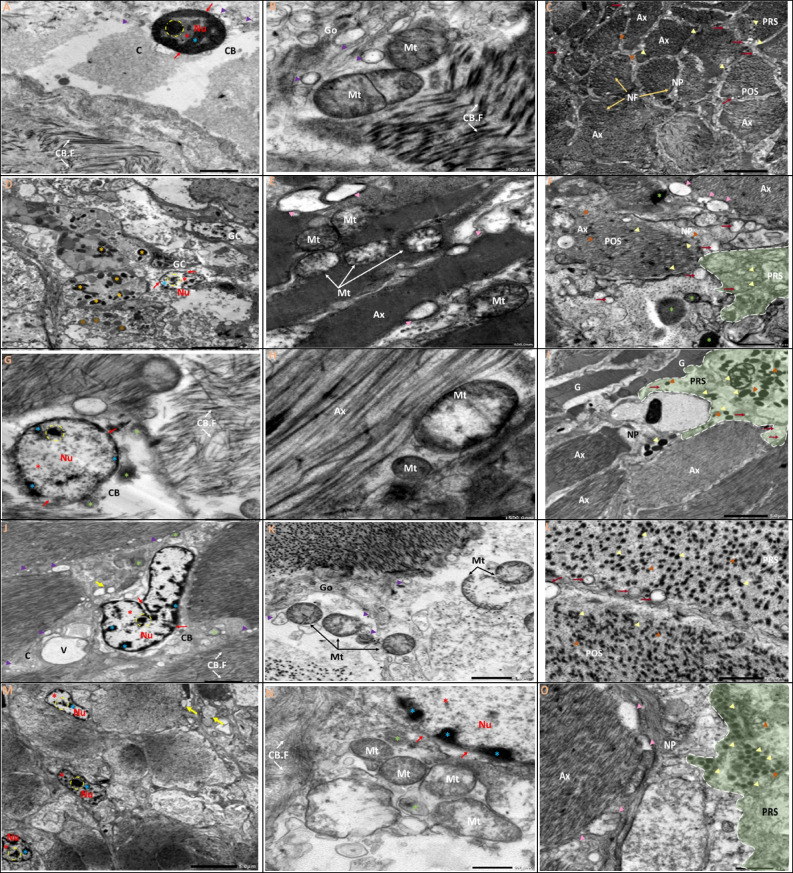

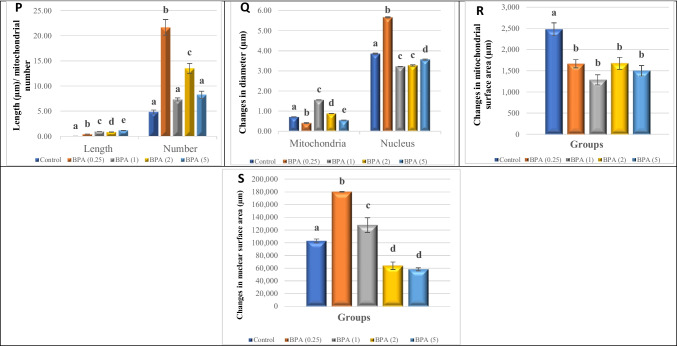
**A**–**O** Transmission electron micrographs of ultrathin sections of the cerebral ganglia of *L. lithophaga*. **A**, **B**, and **C** Control group; **D**, **E**, and **F** 0.25 µg/L BPA-exposed group; **G**, **H**, and **I** 1 µg/L BPA-exposed group; **J**, **K**, and **L** 2 µg/L BPA-exposed group; **M**, **N**, and **O** 5 µg/L BPA-exposed group. Abbreviations and symbols: neuron (Ne), neuropil (NP), nucleus (Nu), nucleolus (dashed circle), cytoplasm (C), neuronal cell body (CB), euchromatin (  ), heterochromatin (  ), nuclear envelope  , cell body filaments (CB.F, white arrows), mitochondria (Mt), golgi complex (Go), pre-synaptic terminal (PRS), post-synaptic terminal (POS), lysosomes (  ), secretory vesicles (V)  , glial cells (GC) → , glial process (G), axon (Ax, white dashed line), axonal neurofilaments (NF)  , clear vesicles  , large dense-core vesicles  , small dense-core vesicles  , autophagosome  , extracellular space  , dense bodies (  ), lipid droplets (  ), degenerated electron-lucent vesicles  , vacuoles (V). **P**–**S** Morphometric analysis of TEM images of cerebral ganglia quantified the length (µm) and number of mitochondria, changes in mitochondrial and nuclear diameter (µm), and changes in surface area (µm) of mitochondria and nucleus, respectively. The scale bars correspond to 2 and 5 µm and 500 nm. Data are depicted as mean ± S.E. of 10 images per group. Statistical analysis was done by ANOVA followed Duncan post hoc multiple comparison test. Different letters on the bars represent statistically significant (*p* ≤ 0.05) differences between control and the four treatment groups

Mussels’ ganglia exposed to 2 µg/L (Figs. 3 and 4G, H) and 5 µg/L (Figs. 3 and 4I, J) BPA showed many drastic degenerative hallmarks, compared to control and other groups, large-sized neurons showed extensive hypercellularity, irregular contour, and some underwent complete degeneration. Small-sized neurons, on the other hand, displayed severe pyknosis, high-frequency apoptosis, and necrosis, as well as the existence of degraded neuron remains. Furthermore, the existence of glial cells, pericellular oedema, and vacuoles, as well as a significantly enlarged and disorganised extracellular space, were observed. Furthermore, high BPA doses damaged the perineurium’s integrity, contributing to the degeneration of axons constructing the neuropile and increasing the rate of hemocyte infiltration, particularly in the CG.

Morphometric quantification of 5 fields with 7 observations per group revealed significant differences in the number of neurons and glial cells, glia-to-neuron ratio, hemocyte infiltration, pyknosis, and vacuolation in the cerebral and visceral ganglia of *L. lithophaga* when compared to controls (Figs. 3 and 4K-D). Figures 3 and 4K show the types of neurons and glial cells in *L. lithophaga* ganglia exposed to BPA. There is a substantial rise in the number of neuronal and glial cells when the two ganglion types are considered (*p* ≤ 0.05, vs. control), with the VG having statistically significant more glial cells than neurons.

We calculated the “glia-to-neuron” ratio using the parameter neurons (Figs. 3 and 4L). There is a trend for slightly fewer glial cells than neurons in the CG and VG, except for the 1 µg/L BPA, where the glia-to-neuron ratio is more balanced.

Furthermore, hemocyte infiltration, pyknosis, and vacuolation of *L. lithophaga* CG and VG ganglia exposed to BPA increased significantly when compared to the control (Figs. 3 and 4M).

### TEM examination

**Fig. 6:**
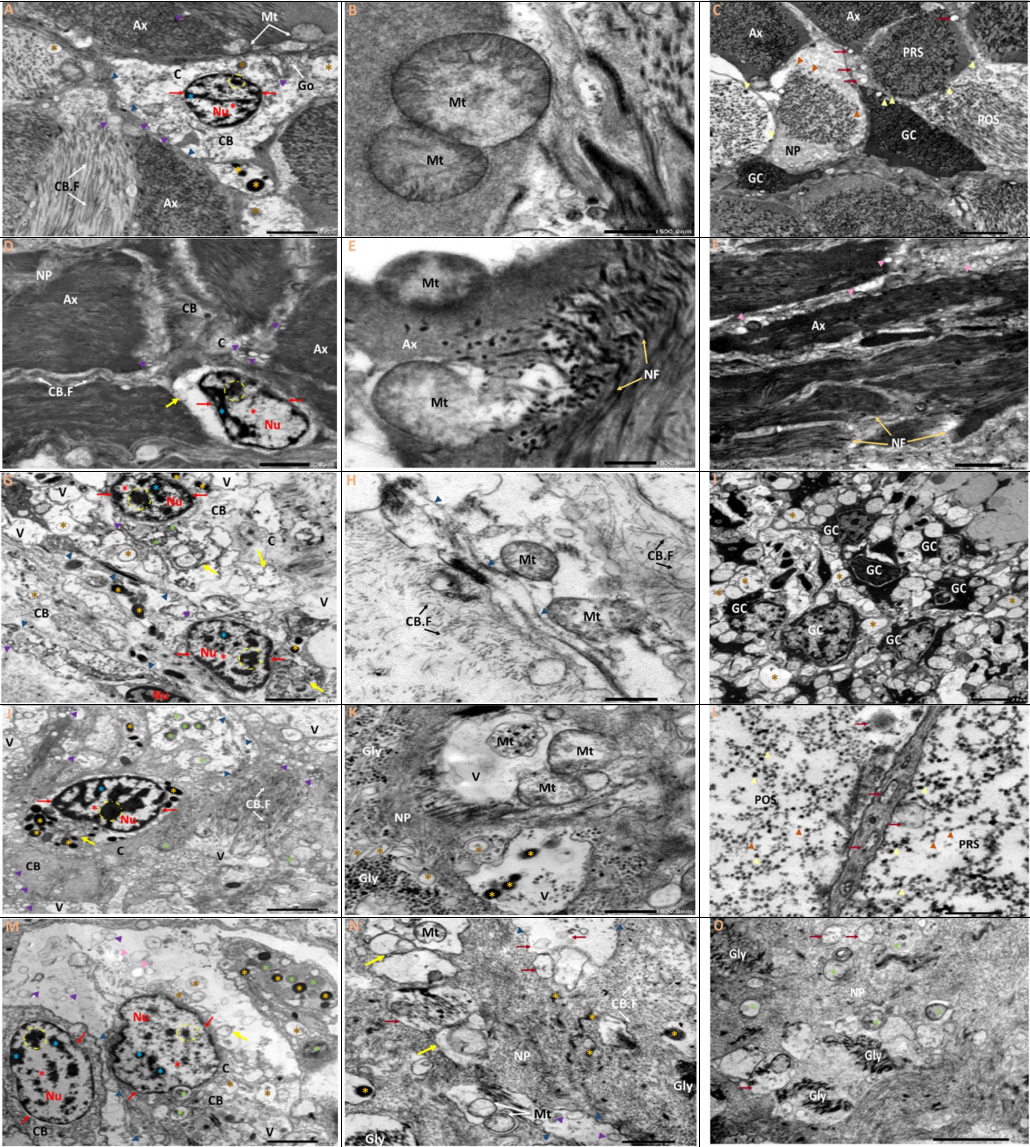

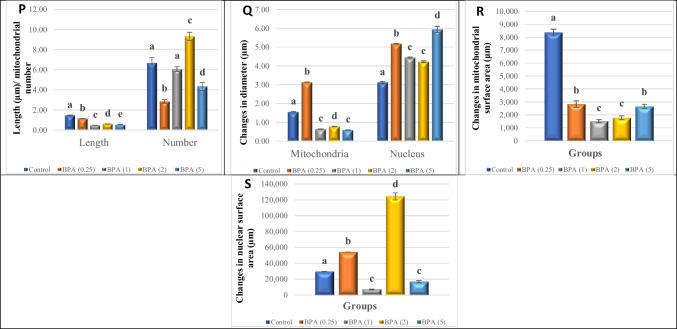
**A**–**O** Transmission electron micrographs of ultrathin sections of the visceral ganglia of *L. lithophaga*. **A**, **B**, and **C** Control group; **D**, **E**, and **F** 0.25 µg/L BPA-exposed group; **G**, **H**, and **I** 1 µg/L BPA-exposed group; **J**, **K**, and **L** 2 µg/L BPA-exposed group; **M**, **N**, and **O** 5 µg/L BPA-exposed group. Abbreviations and symbols: neuron (Ne), neuropile (NP), nucleus (Nu), nucleolus (dashed circle), cytoplasm (C), neuronal cell body (CB), euchromatin (  ), heterochromatin (  ), nuclear envelope  , cell body filaments (CB.F, white arrows), mitochondria (Mt), golgi complex (Go), pre-synaptic terminal (PRS), post-synaptic terminal (POS), lysosomes (  ), secretory vesicles (V)  , glial cells (GC) → , glial process (G), axon (Ax, white dashed line), axonal neurofilaments (NF)  , clear vesicles  , large dense-core vesicles  , small dense-core vesicles  , autophagosome  , extracellular space  , dense bodies (  ), lipid droplets (  ), degenerated electron-lucent vesicles  , glycogen particles (Gly), vacuole (V). **P**–**S** Morphometric analysis of TEM images of visceral ganglia quantified the length (µm) and number of mitochondria, changes in mitochondrial and nuclear diameter (µm), and changes in surface area (µm) of mitochondria and nucleus, respectively. The scale bars correspond to 2 and 5 µm and 500 nm. Data are depicted as mean ± S.E. of 10 images per group. Statistical analysis was done by ANOVA followed Duncan post hoc multiple comparison test. Different letters on the bars represent statistically significant (*p* ≤ 0.05) differences between control and the four treatment groups

TEM and morphometric analysis of the images were used to evaluate the nuclear, mitochondrial, and intracellular morphology of the CG and VG (Figs. 5 and 6). TEM visualisation confirmed the presence of two cell types observed in light microscopy in the two types of ganglia: neurons and glial cells. Because two morphotypes of neurons or nerve cells could be seen in light microscopy, they were classified as large neurons (teardrop cells) and small neurons. Large neurons have a roundish euchromatic nucleus with a prominent nucleolus, and the cytoplasm is characterised by a large number of mitochondria and polyribosomes located along the nuclear membrane. The presence of secretory vesicles that appeared to have sprouted from the Golgi complex was particularly intriguing (Figs. 5 and 6A, B). Small-sized neurons have a large nucleus in the centre, with thin patches of heterochromatin along the nuclear membrane and euchromatin occupying the majority of the nucleoplasm. In comparison to large-size neurons, the cytoplasm is very thin, with well-preserved small mitochondria with longitudinally parallel cristae, free ribosomal clusters, the Golgi complex, and a few lipid droplets. Furthermore, the neuronal cell body filaments appeared to be integrated and intact, as did the extracellular space. Aside from neurons, glial cells seen between cell bodies have elongated nuclei with chromatin clumped at the periphery and a very thin cytoplasm (Figs. 5 and 6A, B). The neuropils of both the CG and VG were examined (Figs. 5 and 6C). It was revealed to be made up of a complex and intricate network of axons. The axons have an axoplasm of medium electron density with a finely granular matrix that is dense with vesicles. The vesicles were divided into two groups based on their size and electron density: clear or electron-lucent vesicles with a finely granulated inner matrix and dense vesicles with a highly osmophilic granule of circular shape. Several synapses with pre- and postsynaptic elements were also found in the neuropils (Figs. 5 and 6C).

The ultrastructural changes in BPA-treated groups revealed different aspects in a dose-dependent manner. The most noticeable ultrastructural finding in the 0.25 µg/L BPA-exposed group was the lightening of the nucleoplasm of the nuclei. The nuclei appeared in the nucleolus with initial fragmentation and dense chromatin at the nuclear periphery and shrank with an irregular nuclear envelope (Figs. 5 and 6D). Results in Figs. 5 and 6E show mitochondria with a partial loss of cristae and devoid of a normal morphological appearance. The existence of autophagosomes, as well as a few lysosomes and small electron-dense granules, indicated autophagy activity in large-sized neurons, notably in the cell bodies of CG neurons. Several electron-dense granules with an enlarged profile are found in nerve fibres. However, some axons showed cytoplasmic texture loss, synaptic vesicle degeneration, slight disintegration of axonal neurofilaments, mitochondrial swelling, and cristae dilatation (Figs. 5 and 6E, F).

The nuclei in the 1 µg/L BPA-exposed group showed lightening of their nucleoplasm, fragmentation in the nucleolus, more chromatin condensation at the nuclear membrane margins, and shrinkage with an irregular nuclear envelope (Figs. 5 and 6G). The cytoplasm was vacuolized, with the appearance of autophagosomes and lipid droplets, particularly in VG neurons (Fig. 6G, I), as well as an increase in the number of lysosomes and electron-dense granules (Figs. 5 and 6G). The mitochondria appeared swollen, with an increased number of cristae that were disorganised (Figs. 5 and 6H). The integrity of cell body filaments was severely compromised in the cellular cytoskeletal system. The nerve fibre alterations were not completely visible in the obtained sections, except for some features in the CG that were similar to those seen in the 0.25 µg/L BPA-exposed group, but the disintegration of the axonal neurofilaments progressed more rapidly (Fig. 5H, I). The neurons of the VG in this group, on the other hand, had an enlarged and disorganised extracellular space, the appearance of activated glial cells with different morphotypes between neuronal cell bodies, and an abundance of lipid droplets (Fig. 5I).

Since these doses are considered high, the 2 and 5 µg/L BPA-exposed groups showed many similarities, with prominent nuclear and cytoplasmic alterations. Indentation, shrinkage, loss of nuclear membrane integrity, withdrawal of peripheral chromatin from the nuclear membrane, and the formation of small electron-dense patches of heterochromatin were observed in the nuclei of large-sized neurons. Furthermore, the nucleoli showed component segregation (Figs. 5 and 6J, M). The most noticeable changes in the cell body were cytoplasmic degeneration and the presence of numerous autophagosomes and lysosomes. The mitochondria grew dramatically in size and complexity, with numerous disorganised cristae. A dilated and disorganised extracellular space containing membranous structures and myelin bodies appeared to separate the neuronal cell bodies (Figs. 5 and 6J, K, M, N). The neuropil contained nerve fibres with degenerated electron-lucent vesicles, a large number of clear synaptic vesicles, some of which contained glycogen particles, and swollen mitochondria with dilated cristae, which were more pronounced in the 5 µg/L BPA-exposed groups (Figs. 5 and 6L, O).

Morphometric quantification of 10 images per group revealed significant differences in the average length and number of mitochondria, as well as the diameter and surface area of the nucleus and mitochondria when compared to controls (Figs. 5 and 6P-S).

## Discussion

Along with its widespread use, BPA is one of the most dangerous environmental pollutants and is frequently detected in aquatic environments (Benjamin et al. 2019; Tang et al. 2020; Miglioli et al. 2021). The current study demonstrated for the first time that changes in the behaviour, antioxidant enzymes, histology, and ultrastructure of *L. lithophaga* ganglia were observed after 28 days of BPA exposure. Behaviour is an important method for determining the toxicity of chemical substances (Zeng et al. 2012). Excurrent and incurrent syphons remained mostly fully open and extended, as did valves in control bivalves. The valves were opened regularly to allow the foot to move actively. The introduction of BPA into the medium caused the first signs of behavioural change. Mussels were observed abruptly closing the valves, indicating a quick response by the animal as part of its protective behaviour against the BPA toxicant. Mussels may have tried to avoid the entry of BPA into the water during filter feeding or respiration because they are filter feeders. The organism’s quick response has been dubbed an “early warning” response (Clotfelter et al. 2004; Brahma and Gupta 2020).

Mov and duration of VC were significantly higher in the BPA-exposed groups; however, the duration of VC is insignificantly lower in the 0.25 and 2 µg/L BPA-exposed groups than in the control. Furthermore, the Mov and FSE were completely stopped in *L. lithophaga* exposed to 5 µg/L BPA. Although the foot is a large organ in mussels that serves as a fleshy anchor during locomotion, research on foot extension and contraction concerning contaminants is limited. Similarly, very few studies have been reported on the behaviour of the extension of syphons in toxic conditions. Bivalves have been shown to close their valves as avoidance behaviour to reduce their exposure to toxic substances (Fournier et al. 2004; Comeau et al. 2017; Vereycken and Aldridge 2023). Foot extension and contraction were reduced or absent in sub-lethal BPA-exposed mussels, which could be attributed to the effect of altered AchE activity caused by the presence of BPA in the test medium. This is consistent with Miserazzi et al. (2020) and our previous studies. It was identified by a considerable increase in the duration of valve closure in mussels exposed to BPA. We propose that decreased neural activity contributed to decreased metabolic activity in muscles, decreased valve activity, and contributed to the catch mechanism via the neuromodulation mechanism (Abd Elkader and Al-Shami 2023). Aiello et al. (1981) found that DA, norepinephrine, epinephrine, and Ach applied to the foot and ganglia of *Mytilus edulis* caused foot contraction and 5-HT caused foot extension. On the other hand, 6-hydroxydopamine’s destruction of dopaminergic nerves prevented foot contraction, whereas 5,6-dihydroxytryptamine’s destruction of serotonergic nerves prevented foot extension. They determined that the CG influenced foot extension and the VG influenced foot contraction. The current findings differ from those of Yasmeen et al. (2012); Brahma and Gupta (2020) in *Lamellidens marginalis*, *Lamellidens jenkinsianus obesa*, and *Parreysia corrugate* in response to toxic substances. In the study of Yasmeen et al. (2012), *L. marginalis* exposed to cadmium tightly closed the shell valves for around the first 12 h and no rapid closure of the shell valves happened later, particularly in high concentrations of cadmium such as Lc50 in different seasons. It was also shown that animals became active after around 24 h of exposure to cadmium Lc0 concentrations and could subsequently open shell valves and extend body parts like those in the control. Due to cadmium shell valves being exposed for 72 h and not being closed. The inhibition of the activity of a calcineurin-like Ca^+2^ regulated phosphatase may be the cause of the persistent opening of the shell valves and loss of the capacity of the adductor muscles to contract following continuous cadmium exposure. Moreover, the movement in Pb-exposed bivalves was dramatically and gradually reduced compared to the control. Furthermore, the movement was completely stopped in 10% of Pb-exposed *P. corrugata*. Foot and syphon extension was completely prevented in 10% Pb-exposed small *L. j. obesa* and both 1% and 10% *P. corrugata*, but only slightly decreased in large *L. j. obesa* (Brahma and Gupta 2020). Behavioural changes in organisms are never constant, and they may exhibit highly collateral and changeable behaviours in response to various types of stress (Bae and Park 2014).

Filter-feeding bivalve molluscs like the *L. lithophaga* mussel may be exposed to BPA in the environment. There is currently little information on the neurotoxicity of BPA on marine bivalve species, particularly in the ganglia tissues of *L. lithophaga*. The findings of this study indicate that lipid peroxidation in the ganglia tissues of *L. lithophaga*, a toxic effect of BPA, appears to be time- and concentration-dependent. Ganglia tissue-specific biomarker determination might provide a deeper insight into the effects of BPA neurotoxicity. Oxidative stress is a critical endpoint in toxicology that has been widely used to investigate the oxidative damage caused by exogenous substances (Aouey et al. 2017). MDA is the end product of lipid oxidation, and its content can be used to indirectly assess the degree of damage to the membrane system. In our study, the MDA content increased significantly at 0.25, 1, 2, and 5 µg/L BPA treatments, which could lead to oxidative stress. A significant increase in MDA concentration can result in lipid peroxidation, which may be due to SOD and CAT’s inability to remove excess reactive oxygen species over time. These findings are consistent with those of Abd Elkader and Al-Shami (2023), who found that *L. lithophaga* had higher MDA levels in the adductor muscles in a concentration-dependent manner. Increased MDA levels indicate oxidative stress and lipid oxidation damage caused by BPA, which can have a deleterious impact on a variety of physiological processes. Furthermore, the mitochondrion respiration chain is dysfunctional because H_2_O_2_ can react with free Fe^2+^ and be transformed into hydroxyl, a reactive radical, via the Fenton reaction. The latter may induce polyunsaturated phospholipid chain oxidation, resulting in lipid peroxidation and functional and structural problems in biological membranes (Ayala et al. 2014; Abd Elkader and Al-Shami 2023). Bivalves were exposed to several types of microplastics, and BPA previously acquired the same results (Ribeiro et al. 2017; Guilhermino et al. 2018; Gnatyshyna et al. 2019; Esperanza et al. 2020).

SOD and other antioxidant enzymes maintain the balance of oxidants and antioxidants in check, protecting cells from oxidative damage (Ren et al. 2013; Zhang et al. 2020). Our findings showed that 0.25 and 2 µg/L BPA exposure strongly stimulated SOD activity in the ganglia of *L. lithophaga*, followed by a significant decrease at 1 and 5 µg/L BPA exposure. These findings suggested that BPA exposure causes an increase in oxidative damage by increasing the production of reactive oxygen species (ROS) and MDA, altering the expression of antioxidant enzyme genes and enzyme activity such as SOD, reducing the ability to scavenge free radicals, and causing severe tissue damage to the ganglia of *L. lithophaga*. This increased enzymatic activity enhanced the removal of ROS generated by either BPA metabolization or electron transport system activity (Almeida et al. 2015). The present results agree with Zhou et al. (2022), who reported an increase in SOD activity, which indicates the generation of superoxide radicals and the development of an adaptation response against polypropylene microplastics in *Tegillarca granosa*. In the study of Uçkun (2022), SOD activity increased in a dose-dependent manner with LC50 and LC50/2 of BPA in crayfish *Astacus leptodactylus*. These results indicate that the antioxidant system is activated in response to BPA exposure (Hemalatha et al. 2015; Uçkun 2022). Furthermore, the increase in SOD activity could be attributed to the ability of intertidal organisms to adapt to environmental changes (McGaw et al. 2015). In some circumstances, ROS levels and antioxidant capabilities are positively correlated with the duration of air exposure (valve closure), suggesting that antioxidant enzymes are activated in response to ROS production. The role of gaping (slight valve opening) in aerobic respiration has been interpreted as a bivalve’s adaptation to long periods of air exposure in the upper intertidal zone. However, for some bivalves found in the subtidal, intertidal, and upper tidal zones, the responses to air exposure are influenced by the environmental conditions they encounter (Yin et al. 2017).

However, SOD activity was inhibited, indicating an imbalance between O_2_^−^ and detoxification of the major antioxidant enzymes (Matozzo et al. 2004). These findings are consistent with those of Abd Elkader and Al-Shami (2023), who demonstrated that BPA inhibits SOD activity, which could be linked to a lack of cellular protective capacity to remove superoxide radicals.

BPA also had a significant impact on the activity of enzymes involved in tissue redox balance maintenance by downregulating the activities of glutathione metabolism enzymes (Canesi et al. 2007). Glutathione and glutathione-related enzymes are effective anti-chemical reactive species defence mechanisms. Total glutathione levels in ganglia decreased in the BPA treatment groups in the current study. Li et al. (2008) previously reported the same results of similar patterns of glutathione level reduction under different profiles of contamination. The results obtained with BPA are consistent with those obtained in mammalian systems, where the xenoestrogen can act as both a pro-oxidant and an antioxidant (Canesi et al. 2007), because BPA has antioxidant activities structurally, while it shows pro-oxidant activity via the estrogen receptor or its metabolites (Kabuto et al. 2003; Canesi et al. 2007). The reason for GSH depletion could be attributed to BPA’s ability to bind to functional cellular groups via nucleophilic substitution reactions through sulfhydryl groups and the detoxification of ROS (Espinosa-Diez et al. 2015). Furthermore, GSH depletion may reduce tissues’ detoxification capacity as well as their susceptibility to oxidative stress (Haque et al. 2019).

AchE is a neurotoxic biomarker of pollution in the environment (Zhang et al. 2020). In this study, AchE activity was reduced by up to 76% compared to controls after 4 weeks of exposure to 0.25, 1, 2, and 5 µg/L BPA. Significantly lower AchE activity in BPA-exposed ganglia tissues suggests that BPA may have negative effects on the cholinergic system of bivalves, such as impaired nervous system function (Haque et al. 2019). This is the first study to show the effects of BPA on AchE activity in *L. lithophaga* ganglia tissues. We hypothesised that the inhibition of AchE caused by BPA was due to their altered affinity for free-SH groups and significant variations in their function, similar to the findings of other studies of Li et al. (2015) and Park et al. (2016). Furthermore, BPA interacts with the aromatic surface of AchE, shrinking the Ach binding space and inhibiting AchE activity (Khazri et al. 2017; Zhang et al. 2020). The reduction in AchE activity confirmed that BPA was neurotoxic to *L. lithophaga*. These findings are consistent with those of Chen et al. (2017), Liu et al. (2019), and Bellas et al. (2022), who found a decrease in AchE activity in zebrafish, *Daphnia magna*, and *Paracentrotus lividus* after BPA exposure. Furthermore, 4-nonylphenol, an endocrine disruptor, is a neurotoxic chemical for mussels, dramatically reducing AchE activity in *Mytilus galloprovincialis* after 30 days of exposure at concentrations ranging from 75 to 100 µg/L (Vidal-Liñán et al. 2015). Additionally, BPA increased myelin expression in the central nervous system and inhibited AchE significantly (Heredia-García et al. 2023).

In molluscs, 5-HT signalling is involved in a variety of key physiological processes ranging from reproduction to neurogenesis and muscle contraction (Kim et al. 2019; Miglioli et al. 2021); as a result, BPA exposure may interfere with the onset of the serotoninergic system, with potentially disastrous consequences in wildlife populations (Miglioli et al. 2021). Our findings showed that BPA treatment caused obvious neurotoxicity, as evidenced by significant decreases in DA and 5-HT levels. Neurotransmitter content in vivo is strictly regulated by corresponding regulatory enzymes (Hermida-Ameijeiras et al. 2004; Jebali et al. 2013). As a result, the inhibition of DA and 5-HT levels observed in this study could be attributed to the simultaneous upregulation of their modulatory enzymes and MAO activity (Gagne and Blaise 2003; Abd Elkader et al. 2021). Furthermore, it is well understood that neurotransmitters exert physiological modulatory functions by binding to specific receptors on target cells (Tang et al. 2020). The results obtained with BPA are consistent with those obtained in mammalian systems, where the decrease in DA may be due to BPA modifying several processes, including DA synthesis, release, and turnover, as well as the expression of both DA transporters and receptors (Saied and Hassan 2014). BPA stimulates 5-HT turnover, resulting in lower 5-HT levels. Moreover, estrogen regulates the number and function of 5-HT receptors, implying that BPA may act through ERs to mediate its action (Xin et al. 2018).

Molluscan invertebrates, especially bivalves, are distinguished by the presence of neuroendocrine cells that emit signalling molecules into the coelomic fluid or hemolymph. As a result, it is possible to suggest that endocrine disturbance in bivalves is mostly neuroendocrine (Canesi et al. 2022). BPA is characterised by its lipophilic composition, which allows it to penetrate the neural tissue of the ganglia and concentrate inside. Accumulation of BPA in neurological tissue causes oxidative stress and activation of several pathways, which may lead to deterioration in both the inter and intracellular structure of neurosecretory cells (Essawy et al. 2021). To date, the mode of action of BPA in the nervous system of mussel bivalves has not been studied at the histopathological and ultrastructural levels, so the current study was also performed to illustrate the histopathological and ultrastructural alterations in the neurons of CG and VG of date mussel (*L. lithophaga*) using dose-dependent concentrations of BPA. Our findings show that both CG and VG have two types of neurons (large and small). Similarly, other researchers have reported size disparities in neurons from different Mytilidae species (McElwain and Bullard 2014; Ruiz-Velasquez et al. 2018). Light microscopic examination of the CG and VG in date mussels treated with various doses of BPA revealed a wide range of cell body alterations in this study. However, higher BPA doses (2 and 5 µg/L) caused more severe damage to neurons and ganglionic architecture. Shrinkage of neuronal cell bodies, eccentric nuclei, an extreme indentation of the nuclear envelope, dissolution of the cytoplasm, degeneration of axon nerve fibres in neuropils, and perineurium disorganisation were all observed in both large and small-sized neurons. Malak et al. (2015) revealed that the neurons of the dorsal root ganglia of streptozocin-treated embryos were less compact, widely spaced, and shrunken. There were empty neural spaces due to nerve cell loss. Gliocytes, or flat epithelial satellite cells, were disorganised and significantly increased in number, with apparent wide perineural spaces. Intracellular space dilation has been observed between neurons in both the cerebrum and the visceral ganglia, which may be attributed to a dysfunction of these cells’ ionic and osmotic balance (Cotran et al. 1999). This is most likely due to the activation of glial cells and their accumulation in clusters to restore neuronal ionic and osmotic balance as they participate in osmoregulation processes. Furthermore, it has been reported that long-term exposure to BPA can induce inflammation and apoptosis in *Cyprinus carpio* by increasing ROS levels and activating distinct signalling pathways, resulting in a significant redox imbalance that could lead to inflammatory responses and apoptosis. This could demonstrate the progression of hallmarks of apoptosis and necrosis, as well as a high frequency of hemocyte infiltration into the ganglionic tissue, particularly in high doses of BPA (2 and 5 µg/L) to phagocyte and eliminate the toxins that existed in the tissues (Schmitt et al. 2012; Elizalde-Velázquez et al. 2023).

Ultrastructural changes in the neural ganglia of the mussel *L. lithophaga* after BPA treatment at various doses might be regarded as neuroplastic or neurodegenerative (destructive). The nuclei showed numerous changes in size and chromatin distribution. They appeared karyolitic, eccentric, and highly shrunken, with nucleoli that were marginated and an irregular nuclear envelope. Bayne et al. (1985) described karyolysis as a late reaction to intoxication in both vertebrates and invertebrates, which is consistent with this finding. Higher BPA doses resulted in an obvious increase in the number of lysosomes and autophagosomes. Autophagy, on the other hand, is a cellular degradation mechanism that is particularly important during developmental stages and under certain environmental stress conditions (Klionsky and Emr 2000). Our ultrastructural findings support the hypothesis that (i) an increase in the number of neuropils and karyolysis activity is a nonspecific reaction of bivalve mollusc neurons to environmental stress factors, and (ii) autophagosomes are involved in mollusc CNS adaptation to environmental stress. As these organelles store the majority of cellular calcium, one of the presumed roles of cytosomes (autophagosomes) is the reversible accumulation and storage of Ca^2+^ ions (Petrunyaka 1982). Recent biochemical and biophysical investigations revealed that several neuromodulators, including 5-HT, Ach, and NO, cause conformational changes in cytosomes (Brazhe et al. 2005; Erokhova et al. 2005). Autophagosomes are involved in several activities that are triggered by neurotransmitters and are related to the redistribution of calcium ions in the cytoplasm during neuron function (Kotsyuba and Vaschenko 2010).

Furthermore, there was an increase in the number of different sizes of electron-dense granules and lipid droplets dispersed throughout the neuronal cell bodies. Similar findings were reported by Fantin and Franchini (1990), who discovered an increase in the number of large and small neurosecretory vesicles in *Viviparus* neurons after being exposed to short-term static pollution by lead, as well as the presence of lipid droplets, both of which are considered remarkable features during intoxication. Scientists have lately focused on mitochondria as organelles involved in the cell’s response to a variety of physiological and environmental stimuli (Kakkar and Singh 2007; Keating 2008). Mitochondrial dysfunction, which is related to higher amounts of ROS and the development of oxidative stress in cells, is at the root of a variety of pathologies, including neurodegenerative disorders in mammals (Mancuso et al. 2007). BPA caused mitochondrial damage in neuronal cell bodies and neuropils. Kim et al. (2003) stated that increased reactive oxygen species production and alteration of mitochondrial membrane permeability, leading to depolarization of the mitochondrial membrane, are the main causes of swollen mitochondria with disintegrated cristae and adenosine triphosphate (ATP) depletion. A growing body of evidence suggests that mitochondrial activities such as Bcl-2 family protein activation, permeability transition pore creation, cytochrome c release, and other apoptosis-inducing factors are involved in apoptotic and necrotic cell death (Fiskum 2001). There has been an increasing interest in the role of oxidative stress as a key component of the stress response in marine species subjected to a variety of harsh environmental variables (Manduzio et al. 2005; Abele et al. 2007; Kotsyuba and Vaschenko 2010).

The release of neurotransmitters from the presynaptic terminal into the synaptic cleft to activate receptors on the postsynaptic neuron is a key component of information transfer in the nervous system. These alterations contribute to the cellular basis of learning and memory in the brain by dynamic recruitment of postsynaptic receptor complexes and modulation of presynaptic vesicular release. Molluscan neuropils contain a wide range of vesicle types. Our findings reveal that the neuropils of both CG and VG are inhabited by a high number of axon terminals containing diverse types of synaptic vesicles (clear, tiny dense-core, and big dense vesicles). The ultrastructurally distinct forms of synaptic terminals identified in this study may indicate different neurotransmitter contents, according to the concept that each vesicle type could contain a different transmitter. This is consistent with Mahmud et al. (2007), Essawy et al. (2009), and Kotsyuba and Vaschenko (2010). Neuropeptides, like biogenic amines, are released in either the CNS or peripheral nervous system neuropils (Sinakevitch et al. 2018). Neurotransmitters are involved in several behaviours in bivalves, but their diversity and location in these creatures’ neural systems are unknown (Kotsyuba et al. 2020). Neuromodulators, in this sense, are the chemical substrates that underpin plasticity in all neurological systems. Their field of action extends beyond the neurological system. Neuromodulators orchestrate a slew of neuronal and physiological processes that, when combined, can serve a specific behavioural context or physiological condition, and reciprocal interactions between the nervous system and metabolic or physiological states in non-nervous tissues are widely recognised as a new research focus (Sinakevitch et al. 2018).

Neuronal processes are known to contain a large number of mitochondria, and their deficit can result in structural and functional damage to dendrites, axons, and their terminals (Mattson 2007). Energy depletion and/or increased oxidative damage to numerous synaptic proteins linked to mitochondrial dysfunction can cause a local dysregulation of calcium homeostasis and synaptic degeneration without cell death (Kotsyuba and Vaschenko 2010). Loss of cytoplasmic texture, degeneration of synaptic vesicles, disruption of myelin bodies, and swelling of mitochondria with dilatation of their cristae were the most common changes observed in the neuropils of both ganglia. BPA’s disruption of axonal transport may deplete newly formed synaptic vesicles and promote synaptic terminal degradation, leading to dysfunctions of the neurons that make up the ganglia. Long-term BPA exposure in adult mice reduced synaptic density and altered the structure of the synaptic interface, including enlargement of the synaptic cleft and decreased active zone and post-synaptic terminal thickness, according to Xu et al. (2013).

Furthermore, it is well known that neurons can accumulate membranous bodies under any stress (Ryan and Fawzi 2019). This may occur because cellular organelles go through a specific process of destruction and digestion, and as a result, undigested material accumulates as membranous residual bodies. The ultrastructure of CG and VG, on the other hand, revealed a significant increase in clear vesicles in the cytoplasm of neuronal cell bodies and nerve terminals. Furthermore, these terminals demonstrated a significant increase in glycogen granule aggregation. In the current study, however, there was an increase in glycogen granules following high doses of BPA exposure, particularly in VG. This could be because glycogen is present in a metabolically inert form with no bound phosphorylase required for breakdown, as this is considered a part of a long list of metabolic changes that are consequences of such intoxication (Pipe 1987).

## Conclusions

BPA exposure at environmentally plausible concentrations has been shown to produce significant neurotoxic effects on the ganglia of *L. lithophaga*, which has never been reported before. Because BPA has the potential to accumulate, induce oxidative stress, and cause behavioural, neurochemical, histological, and ultrastructural changes, it should be studied further in the future. Given the neurological significance of AchE, DA, and 5-HT, as well as the significance of observed alterations, attempts to link enzyme activity and neurotransmitter levels with ecologically important behavioural endpoints are warranted. However, because behaviour can influence how much the biomarker is affected, the ability of bivalves to modify their BPA exposure through valve closure is an important factor to consider. These biochemical deficits influence cellular architecture and size, as well as nucleus and mitochondria morphology, distribution, size and function.

## Data Availability

Data will be made available on request.
